# T cell immune response predicts survival in severely ill COVID-19 patients requiring venovenous extracorporeal membrane oxygenation support

**DOI:** 10.3389/fimmu.2023.1179620

**Published:** 2023-08-01

**Authors:** Zsuzsanna Ulakcsai, Liliana Szabo, Zsofia Szabo, Eva Karaszi, Tamas Szabo, Levente Fazekas, Alexandra Vereb, Nora Fanna Kovacs, Dora Nemeth, Eniko Kovacs, Endre Nemeth, Gyorgy Nagy, Hajnalka Vago, Bela Merkely

**Affiliations:** ^1^ Heart and Vascular Center, Semmelweis University, Budapest, Hungary; ^2^ William Harvey Research Institute, Queen Mary University of London, London, United Kingdom; ^3^ Department of Laboratory Medicine, Semmelweis University, Budapest, Hungary; ^4^ Pediatric Healthcare Center, Council of Budafok-Tétény, Budapest, Hungary; ^5^ Department of Rheumatology and Clinical Immunology, Semmelweis University, Budapest, Hungary; ^6^ Department of Genetics, Cell- and Immunobiology, Semmelweis University, Budapest, Hungary; ^7^ Hospital of the Hospitaller Order of Saint John of God, Budapest, Hungary; ^8^ Department of Sports Medicine, Semmelweis University, Budapest, Hungary

**Keywords:** SARS-COV-2, COVID-19, ECMO, immune dysregulation, serological test, antibody response, T cell response

## Abstract

**Introduction:**

There is a critical gap in understanding which SARS-CoV-2 patients would benefit most from venovenous extracorporeal membrane oxygenation (VV-ECMO) support. The potential role of a dysregulated immune response is still unclear in this patient population.

**Objectives:**

To assess the potential predictive value of SARS-CoV-2 specific cellular and humoral immune responses for survival in critically ill COVID-19 patients requiring VV-ECMO.

**Methods:**

We conducted a prospective single-center observational study of unvaccinated patients requiring VV-ECMO support treated at the intensive care unit of Semmelweis University Heart and Vascular Center between March and December 2021. Peripheral blood samples were collected to measure the humoral and cellular immune statuses of the patients at the VV-ECMO cannulation. Patients were followed until hospital discharge.

**Results:**

Overall, 35 COVID-19 patients (63% men, median age 37 years) on VV-ECMO support were included in our study. The time from COVID-19 verification to ECMO support was a median (IQR) of 10 (7-14) days. Of the patients, 9 (26%) were discharged alive and 26 (74%) died during their hospital stay. Immune tests confirmed ongoing SARS-CoV-2 infection in all the patients, showing an increased humoral immune response. SARS-CoV-2-specific cellular immune response was significantly higher among survivors compared to the deceased patients. A higher probability of survival was observed in patients with markers indicating a higher T cell response detected by both QuantiFeron (QF) and flow cytometry (Flow) assays. (Flow S1 CD8+ ≥ 0.15%, Flow S1 CD4+ ≥ 0.02%, QF CD4 ≥ 0.07, QF whole genome ≥ 0.59). In univariate Cox proportional hazard regression analysis BMI, right ventricular (RV) failure, QF whole genome T cell level, and Flow S1 CD8+ T cell level were associated with mortality, and we found that an increased T cell response showed a significant negative association with mortality, independent of BMI and RV failure.

**Conclusion:**

Evaluation of SARS-CoV-2 specific T cell response before the cannulation can aid the risk stratification and evaluation of seriously ill COVID-19 patients undergoing VV-ECMO support by predicting survival, potentially changing our clinical practice in the future.

## Graphical Abstract

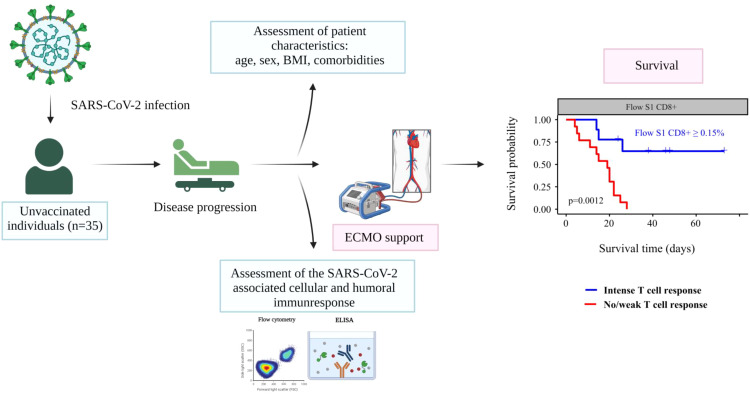

## Introduction

Coronavirus disease 2019 (COVID-19) caused by the SARS-CoV-2 virus has resulted in approximately 6.5 million deaths globally as of December 2022. Although most COVID-19 patients have moderate symptoms and recover without complications, some develop severe respiratory failure requiring intensive care unit (ICU) admission and, often, invasive mechanical ventilation (1, 2). Approximately 5–7% of COVID-19 patients are considered critically ill, showing lung failure and acute respiratory distress syndrome (ARDS). In severe ARDS, when mechanical ventilation cannot maintain adequate oxygenation and/or CO2 elimination, venovenous extracorporeal membrane oxygenation (VV-ECMO) can be considered to support gas exchange and minimize ventilator-induced lung injury (3, 4).

The SARS-CoV-2 virus mainly enters the host cell by binding to the angiotensin-converting enzyme 2 (ACE2) receptor. This process can be blocked by the neutralizing antibody, provided that it binds to the receptor binding domain of the S1 protein (5). However, quite the opposite course of events could be observed in severe COVID-19 cases (6). In critically ill COVID-19 patients, the initial response to SARS-CoV-2 infection is characterized by major dysfunction of the COVID-specific immune response, which is associated with organ damage *via* neutrophil myeloperoxidases, other proteinases, and the excessive production of proinflammatory cytokines (IL-6, TNF-α).

Early research has highlighted a potential connection between the functionality of SARS-CoV-2-specific T cells and patient outcomes. A study by Sattler et al. (7), for instance, found that deceased individuals were significantly more likely not to have a cellular response to SARS-CoV-2 proteins. These findings suggest that the body’s immune response, particularly the T cell response, may play a crucial role in determining the severity and outcome of the disease. However, despite these early insights, the full extent and duration of the immune response to SARS-CoV-2 remain largely unexplored (8, 9). In cases of severe SARS-CoV-2 infection leading to ARDS, which typically develops within 8-12 days, it is thought that both host cell-derived substances and direct viral effects play a role. Yet, a clear pathomechanical link between the initial immune response in critically ill COVID-19 patients and their clinical outcome remains elusive.

At the onset of the pandemic, our understanding was limited by a paucity of research into the pathomechanism of the disease, its clinical trajectory, and effective therapeutic strategies. During this early phase, ECMO support, despite its considerable financial, human resources, and technical requirements, emerged as a potential intervention for patients with severe respiratory distress. The ECMO support functioned as a last chance for severely ill patients, serving as their only hope for survival. However, initial findings indicated that ECMO had a high mortality rate and limited effectiveness (10). Contemporary reports suggest a higher survival rate, attributed to the application of enhanced pre-cannulation protocols, which have been refined based on the accumulated clinical evidence (11). Despite these advances, a critical knowledge gap persists: it remains unclear which patients will benefit from ECMO support and which will not.

Several conventional risk factors, including older age, male sex, chronic lung disease, delayed cannulation, and extended duration of invasive mechanical ventilation (IMV), have been associated with worse outcomes in severely ill SARS-CoV-2 patients requiring ECMO support (12). Additionally, Ramanathan et al. found that a longer duration of ECMO was linked with increased mortality in this patient population (4). However, these established factors only account for a portion of the observed variability in patient prognoses, suggesting that other, yet unaccounted elements may influence the outcomes.

We hypothesize that such elements might be found in the immune response; specifically, individual variations in T cell response to SARS-CoV-2 could potentially account for the variance observed in patient outcomes. If proven, these immune response markers might serve not only as prognostic indicators but also as therapeutic targets, opening up new avenues for future research. Therefore, our study aims to delve deeper into the peculiarities of the dysregulated immune response to SARS-CoV-2 infection in critically ill COVID-19 patients requiring VV-ECMO support. We propose to investigate the incremental prognostic information conveyed by markers of a dysregulated immune response in addition to others such as conventional risk factors and comorbidities.

## Methods

### Study population and setting

**Graphical abstract** gives a visual overview of our study. We conducted a prospective single-center observational study of critically ill patients treated at the intensive care unit of Semmelweis University Heart and Vascular Center between 1 March and 31 December 2021. The inclusion criteria for enrolment into the study were: 1) age >18 years, 2) laboratory-confirmed SARS-CoV-2 infection (by real-time-PCR test), and 3) initiation of VV-ECMO support due to refractory COVID-19 ARDS. All the patients included in our study were classified as severely ill (D10) according to the World Health Organization’s (WHO) criteria as of 27^th^ May 2020 (13). We excluded all anti-SARS-CoV-2 vaccinated patients (n=2).

### Ethics statement and patient involvement statement

Ethical approval was obtained from the National Public Health Center under the ethical standards laid out in the 1964 Declaration of Helsinki and its later amendments (IV/2568-1/2021/EKU). All participants or their legal guardians gave their written informed consent for participation in the analysis. It was not possible to involve patients or the public in the process of designing, conducting, reporting, or dissemination plans of our research.

### Clinical protocol/ICU care protocol

We followed the Extracorporeal Life Support Organization COVID-19 Interim Guidelines updated recommendations during the indication of the ECMO support (14). All intensive care units in Hungary followed the COVID-19 Treatment Guidelines for pharmacological therapy during the study period. While all care-providing hospitals adhered to the current guidelines for COVID pneumonia, there were natural variances in clinical experience and the clinical course of the disease over time. Patients were referred to our tertiary and regional ECMO center with verified pulmonary organ failure and no other end-organ failure was documented prior to therapy initiation. All patients in the initial care facility received antiviral medication (remdesivir 100 mg daily for 5 days) and glucocorticoid (dexamethasone 0.1 mg/kg daily for 3 days, with a daily maximum dose of 8 mg, which was then tapered over the next 4 days). Upon arrival, all patients were urgently cannulated and VV-ECMO treatment was initiated. The preferred cannulation strategy involved the use of the right femoral vein for drainage (with a long cannula) and the right jugular vein for reinfusion (with a short cannula). The CytoSorb absorber, which can lower circulating pro- and anti-inflammatory cytokines, potentially improving disease course and outcome (15), was utilized in all cases. Lung protective mechanical ventilation was established for all the patients. We assessed the levels of prealbumin, IgA, IgG, and IgM from blood samples taken at the time of ECMO cannulation. If IgM was <0.4 g/L, we administered Pentaglobin, a commercially available IgM- and IgA-enriched immunoglobulin formulation (12% IgM, 12% IgA, and 76% IgG). Patients received an initial bolus at a rate of up to 0.6 mL (30 mg)/kg/h for 6 hours, followed by a continuous maintenance rate of 0.2 mL (10 mg)/kg/hour for 72 hours (total dose ≥0.9 g/kg).

As patients were admitted to our ward, referral laboratory test results, risk factors, and anthropometric data were compiled. During ECMO cannulation, blood samples were collected from all patients for immunological tests and SARS-CoV-2 PCR, confirming viremia in all cases. Respiratory/nasopharyngeal samples were only taken at later stages from patients undergoing therapeutic bronchoscopy, with PCR tests consistently returning positive results. Each patient underwent a comprehensive initial clinical assessment that included a full laboratory test, transesophageal echocardiography, and abdominal ultrasound scan. Microbiological samples were obtained from deep airways (bronchoscopy was utilized) if the PaO2/FiO2 ratio worsened. Once hemodynamic stability was achieved, patients were reassessed every 24 hours following the same protocol. Patient follow-up continued until hospital discharge. The primary endpoint of our study was all-cause mortality during hospitalization.

### Markers of the COVID-19 immune response

At the VV-ECMO cannulation, prior to therapy initiation, dedicated peripheral blood samples were collected to measure the humoral and cellular immune status of the patients. **Supplementary Table 1** provides detailed documentation of the markers of humoral and cellular immune responses measured in our study. In summary, we obtained comprehensive information about the humoral immune response against SARS-CoV-2 by measuring IgG, IgA, and IgM antibody levels specific to the SARS-CoV-2 spike (S) protein receptor binding domain (RBD) based on the double-antigen sandwich principle using electrochemiluminescence (16). Furthermore, we also examined the antibodies produced against different antigens of the virus; nucleocapsid and spike proteins using dedicated enzyme-linked immunosorbent assay (ELISA) tests, which assess the appearance of different isotypes of antibodies (17). First, the human antibodies of the immunoglobulin class IgG and IgA against the S1 domain of the spike protein of SARS-CoV-2 in serum or plasma. Second, the antibodies of the immunoglobulin class IgG and IgM against modified nucleocapsid protein (NCP) of SARS-CoV-2 in serum or plasma.

We also ascertained the cellular immune response of the study participants. For this purpose, we applied interferon-gamma release assays (IGRA) and flow cytometry. QuantiFERON (QF) SARS-CoV-2 ELISA test is based on the production of interferon-gamma (IFN-γ) by lymphocytes in peripheral blood. The collection tubes consist of three antigen tubes: Ag1, Ag2, and Ag3. These use a combination of antigens specific to SARS-CoV-2 to stimulate lymphocytes. We used blood collection tubes coated with specific SARS-CoV-2 peptides pool from the spike antigen (S1 S2 RDB) in case Ag1 (CD4+) and Ag2 (CD4+- CD8+) were present and blood collection tubes coated with specific SARS-CoV-2 peptides pool from the spike and additional peptides issued (N (nucleocapsid) and M (M protein) domains) from the full genome of the SARS-CoV-2 virus in case Ag3 was present. After 16-24 hours of incubation at 37°C, the IFN-γ produced during the cell activation was measured from the separated plasma samples (18). In our study, for transparency, results from Ag1 quantification are referenced as ‘QF CD4+’, from Ag2 as ‘QF CD4+ and 8+’, and from Ag3 as ‘QF whole genome’.

Virus-specific T cells were detected by functional flow cytometry based on IFN-γ secretion using peptivators of both spike (S1) and nucleocapsid (NC) antigens for T cell stimulation. T cell activation, staining, and analysis were performed according to Miltenyi Biotec protocols tailored to the local equipment and personnel. The ratio of INF-γ positive virus-specific T cells was determined within the CD4+ and CD8+ T cell population separately, compared to negative control. **Supplementary Figure 1** shows the flow cytometry gating strategy analysis plot. Results are referenced in this study as “Flow S1 CD4+”, “Flow S1 CD8+” and “Flow NC CD4+”, “Flow NC CD 8+”.

### Statistical analysis

All statistical analyses and figure creation were carried out using the R programming language (v4.0.4) and the Medcalc statistical program. We report the data completeness in **Supplementary Table 2**. Variables with over 40% missingness were removed from further analysis. Wilcoxon signed-rank test was used to assess the statistical significance of the difference between the results of antibody and T cell reactivity assays, as well as hematological parameters among patients stratified by survival. Correlations between the measures of immune response to SARS-CoV-2 infection were ascertained using Spearman rank correlation. Survival analysis was supported by the Survminer package and the p-values for the difference between Kaplan–Meier curves were calculated using the log-rank test. Optimal cutoff values for predicting the survival of patients based on the measured immunological features were calculated using the cut-point R package. ROC analysis was carried out using the plotROC library. The impact of clinical variables, including age, sex, comorbidities, time spent on ECMO support, and immune response to SARS-CoV-2 on patient survival was assessed using Cox proportional-hazards models. Variables showing positive associations during the univariate test were candidates for the multivariate analysis; we report two separate models for highly correlated variables.

## Results

### Baseline characteristics

Overall, 35 severely ill COVID-19 patients (63% men, median age 37 years) on VV-ECMO support were included in our study. The baseline characteristics of the study population are depicted in **Table 1**. All patients were unvaccinated, none had prior SARS-CoV-2 infection or any previous symptoms indicating SARS-CoV-2 infection. Overall, high-risk factor burden was reported among the patients: 49% (17/35) obesity, 29% (10/35) hypertension, 11% (4/35) diabetes, and 9% (3/35) current smoking. Moreover, chronic obstructive pulmonary disease (n=2) and hypothyroidism (n=2) were reported as well as one patient each with atrial fibrillation, myocardial infarction, Hashimoto’s thyroiditis, and arthritis.

**Table 1:** Baseline characteristics.

	All Patients (n=35)	Survivors (n=9)	Deceased (n=26)	P value
Male (%)	62.9% (22/35)	66.7% (6/9)	61.5% (16/26)	0.787
Female (%)	37.1% (13/35)	33.3% (3/9)	38.5% (10/26)	
Age (years) median (IQR)	37 (32-49)	34 (28-42)	39 (32-49)	0.250
Body Mass Index (kg/m^2^)	29.5 (27.8-34.5)	29.2 (27-30.1)	30.9 (27.8-37)	0.070
Obesity	48.6% (17/35)	22.2% (2/9)	57.7% (15/26)	0.071
Hypertension	28.6% (10/35)	22.2% (2/9)	30.8% (8/26)	0.630
Diabetes	11.4% (4/35)	22.2% (2/9)	7.7% (2/26)	0.245
Smoking	8.6% (3/35)	0% (0/9)	11.5% (3/26)	0.294
Atrial fibrillation	2.9% (1/35)	0% (0/9)	3.8% (1/26)	0.556
Myocardial infarction	2.9% (1/35)	11.1% (1/9)	0% (0/26)	0.089
Chronic obstructive pulmonary disease	5.7% (2/35)	0% (0/9)	7.7% (2/26)	0.398
Hypothyroidism	5.7% (2/35)	0% (0/9)	7.7% (2/26)	0.398
Hashimoto’s thyroiditis	2.9% (1/35)	0% (0/9)	3.8% (1/26)	0.556
Arthritis	2.9% (1/35)	0% (0/9)	3.8% (1/26)	0.556

Baseline demographic and clinical characteristics and history at intensive care unit admission. Categorical variables are given in % (n/total n), and continuous variables are given in median(interquartile range).

### ECMO cannulation and initial assessment

Patient characteristics at the initial assessment after ECMO cannulation are summarized in **Table 2**. The time from COVID-19 verification and invasive mechanical ventilation to ECMO support was a median (IQR) of 10 (7-14) and 2 (1-5) days, respectively. Upon the assessment in our institution within 24 hours of the ECMO initiation, 26% of patients had renal failure, and continuous renal-replacement therapy was immediately started. Transesophageal echocardiography showed that 49% of patients had right heart failure, therefore inotrope therapy was initiated. Furthermore, based on the samples taken during bronchoscopy, 49% of the patients showed bacterial superinfection, which was treated with targeted antibiotics.

**Table 2:** Patient characteristics at the initial assessment after ECMO cannulation.

	All Patients (n=35)	Survivors (n=9)	Deceased (n=26)	P value
Time from COVID-19 symptoms to ECMO (days)	10 [7–14]	12 [9-14]	9.5 [7-14]	0.257
Time from IMV to ECMO (days)	2 [1-5]	3 [1-5]	2 [1-3]	0.701
Time on ECMO support (days)	13 [9-19]	12 [11-23]	14 [9-19]	0.763
Hospitalization (days)	20 [14-28]	46 [43-74]	17 [11-20]	<0.001
*Renal failure prior to ECMO cannulation	25.7% (9/35)	22.2% (2/9)	27.0% (7/26)	0.784
*Right ventricular failure prior to ECMO cannulation	48.6% (17/35)	11.1% (1/9)	61.5% (16/26)	0.01
*Liver failure prior to ECMO cannulation	0% (0/35)	0% (0/9)	0% (0/26)	NA**
*Bacterial superinfection prior to ECMO cannulation	48.6% (17/35)	55.6% (5/9)	46.2% (12/26)	0.632
White Blood Cell	12.61 [9.92 -13.89]	11.3 [6.1-13.9]	12.9 [9.3-16.9]	0.227
Lymphocyte count	0.93 [0.70 -1.36]	0.7 [0.5-1.4]	1.1 [0.7-1.5]	0.235
Lymphocyte percentage	9.2 [7.02 -10.3]	9.2 [4.6-11.9]	9.2 [6-11]	0.88
CRP (mg/L)	113.9 [38.9 – 216.4]	66.2 [14.2 – 110.1]	146 [42 – 226.8]	0.07
LDH (U/L)	661 [506 – 960]	625 [472.5 – 976.3]	676 [498 – 946]	0.777

Comparison between surviving and deceased participants. Categorical variables are given in % (n/total n), and continuous variables are given in median [interquartile range]. IMV, invasive mechanical ventilation.

*Diagnosed as per the initial assessment performed in our institution, but unknown at the time of ECMO cannulation. **Not applicable.

### Follow-up and mortality

Of the 35 ECMO patients, 9 (26%) were discharged alive and 26 (74%) died during their hospital stay. A pairwise comparison between surviving and deceased patients showed no difference in terms of risk factor burden, comorbidities, initial laboratory assessment, or drugs received in the intensive care unit (**Tables 1**, **2**). Survivors were hospitalized for a significantly longer time compared to deceased study participants. In contrast, there was no difference in the time delay from COVID-19 symptoms to ECMO or the time spent on IMV before ECMO conversion between surviving and deceased patients. ECMO support lasted for a median (IQR) of 13 (3-29) days and we observed no difference between the two groups. Drug therapies used during ECMO support, including vasopressors, inotropes, and antibiotics, are presented in **Table 3**. Immuno-modulators given as an additional therapy were administered at a later stage, once the results were known.

**Table 3:** Patient characteristics during ECMO support.

	All Patients (n=35)	Survivors (n=9)	Deceased (n=26)	P value
Renal failure after ECMO cannulation	45.7% (16/35)	22.2% (2/9)	54% (14/26)	0.106
New onset liver failure after ECMO cannulation	14.3% (5/35)	0% (0/9)	19% (5/26)	0.161
Bacterial superinfection after ECMO cannulation	100% (35/35)	100% (9/9)	100% (26/26)	0.004
Multi-organ failure after ECMO	80% (28/35)	22.2% (2/9)	100% (26/26)	<0.001
Vasopressor	100% (35/35)	100% (9/9)	100% (26/26)	0.004
Inotrope	48.6% (17/35)	11.1% (1/9)	61.5% (16/26)	0.01
Pentaglobin	37.1% (13/35)	55.6% (5/9)	30.8% (8/26)	0.191
Antibiotics	48.6% (17/35)	55.6% (5/9)	46.2% (12/26)	0.632

Comparison between surviving and deceased participants. Categorical variables are given in % (n/total n), and continuous variables are given in median [interquartile range]. IMV, invasive mechanical ventilation.

Human normal immunoglobulin: Pentaglobin (Biotest Pharma GmbH) ATC: J06B A02 (IgG, IgM, IgA).

Antibiotics: targeted therapy based on microbiological evaluation.

Baseline laboratory assessment of white blood cell (WBC) count and lymphocyte count or percentage and inflammatory markers (neutrophil count, CRP, and LDH) showed no difference between surviving and deceased patients (**Table 2**). During ECMO support, we observed the following complications: renal failure occurred in 46% of patients and liver failure manifested in 14%. Importantly, all patients (100%, 35/35) developed superinfection, and 80% (28/35) experienced multiorgan failure during their stay (**Table 3**).

### Immune response

**Figure 1:**
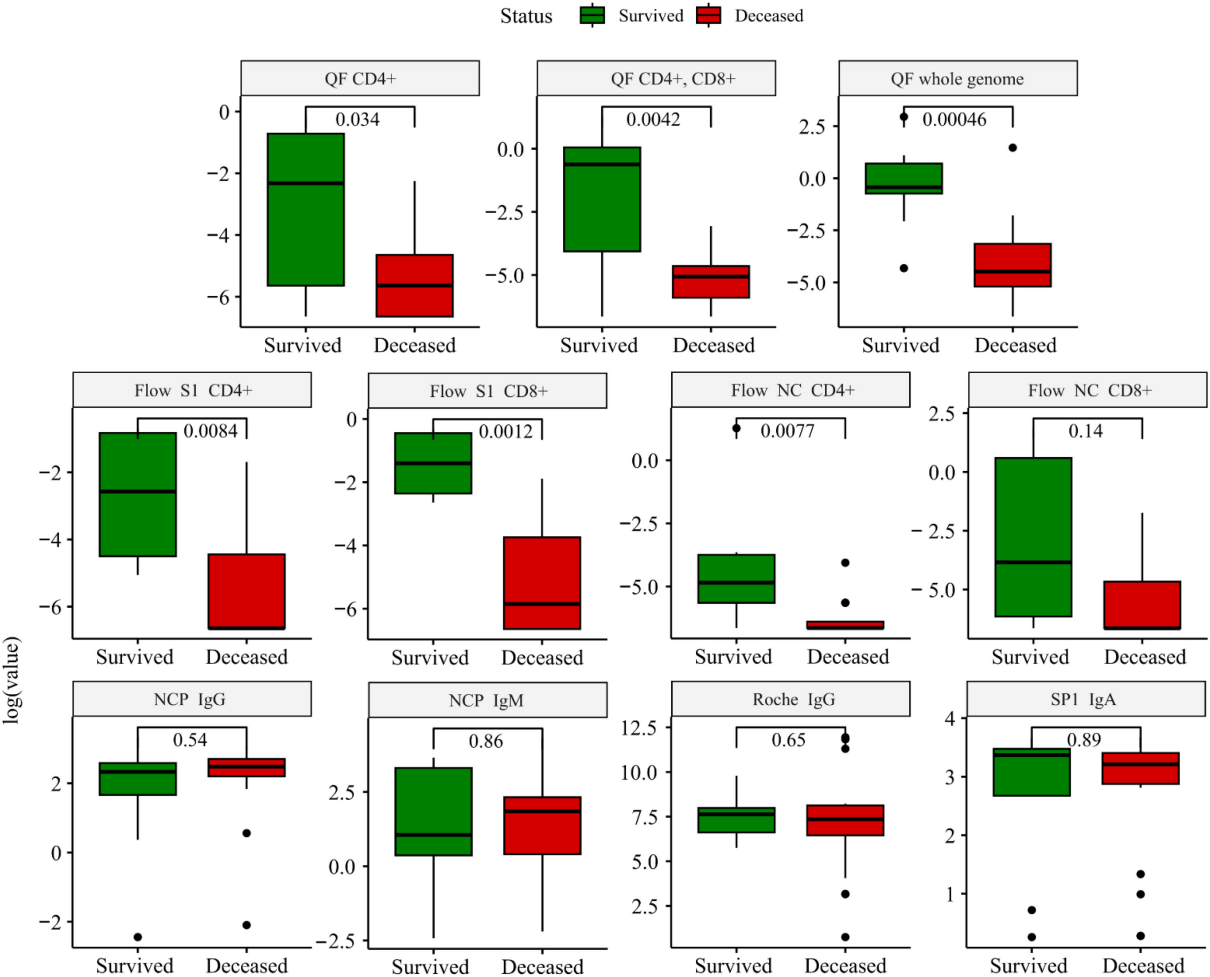
Boxplots depicting the comparison between survivors (green) and deceased (red) patients. The comparison was performed using the Wilcoxon test, and immune markers are shown on a logarithmic scale to account for the skewness of the data. NC, nucleocapsid; SP, spike protein; QF, quantiferon. The cutoff value for each immune marker: QF: >0.15 IU/ml; Roche IgG: >0.8 U/ml; Flow: >0.02%; SP1 IgA >1.1 U/ml; NCP IgM: >1.1 U/ml – positive. A detailed description of the applied methodology and grading system is found in **Supplementary Table 1**.

Overall, immune tests confirmed ongoing SARS-CoV-2 infection in all the patients, showing accelerated humoral immune response as per the Roche IgG [163(87-281) U/ml; positive: >0.8 U/ml] or SP1 IgA [10(7-11); positive: >1.1] levels. We compared the immune response, measured before ECMO initiation of surviving and deceased patients (**Figure 1**). There was no difference among these groups in the markers of the humoral immune response. In contrast, the SARS-CoV-2-specific cellular immune response was significantly higher among survivors compared to deceased patients. This was true for three out of four parameters assessed by Flow Cytometry (S1 reactivity of both CD4+ and CD8+ T cells, and also nucleocapsid reactivity of CD4+ T cells) as well as for all antigen reactivity in QuantiFeron-like assays (**Figure 1**).

**Figure 2:**
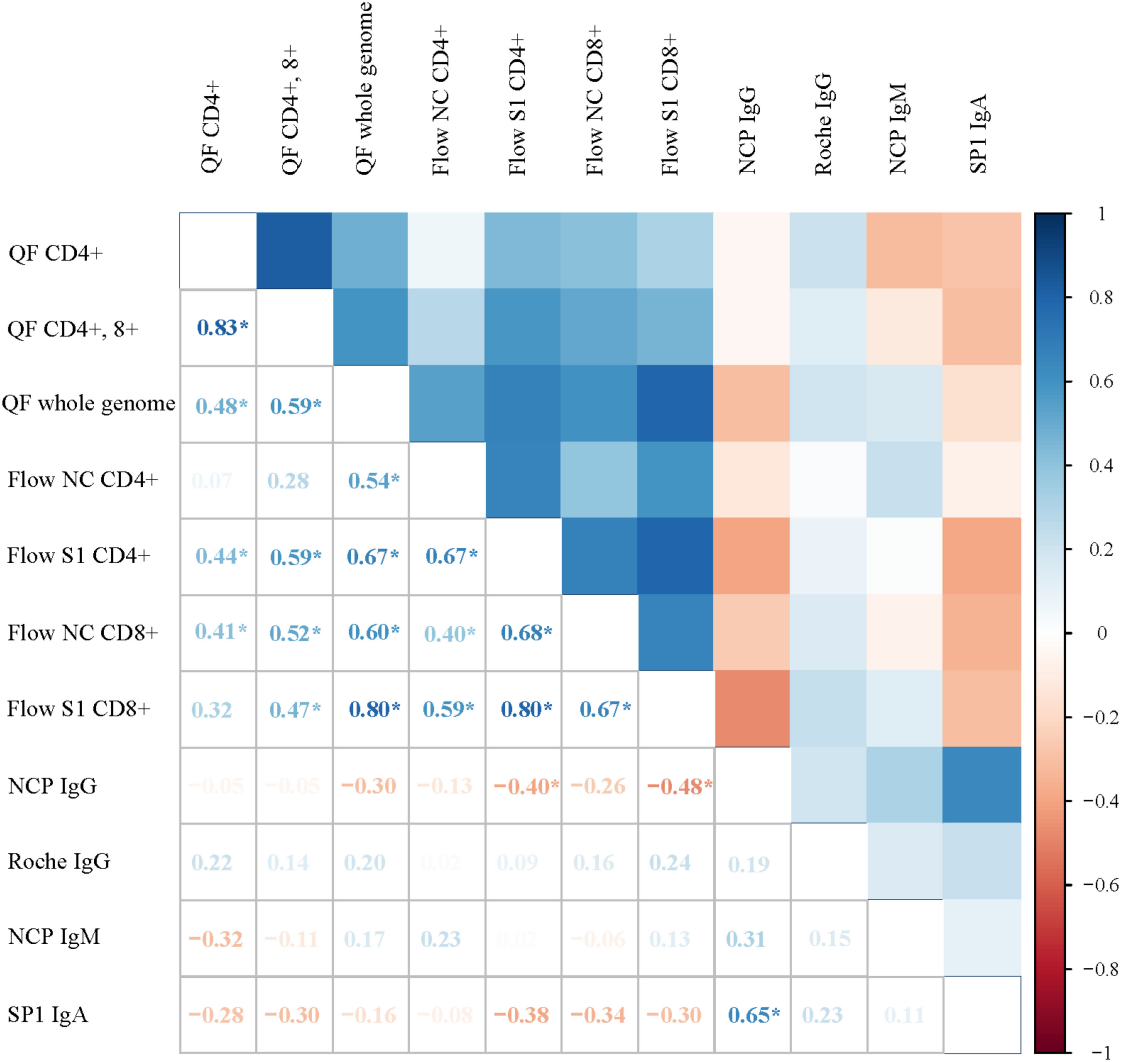
Correlation matrix showing the associations between markers of the SARS-CoV-2 immune response among critically ill COVID-19 patients. Color-coded boxes in the upper part of the figure illustrate the strength of the correlation between immune markers, whereas Rho values calculated from Spearman rank correlation are shown in the lower segment. Each box depicts the results from correlations; positive values signify positive correlations and negative values show negative correlations. Asterix (*) shows significant correlations.

We assessed the correlations of the immune markers. We found a strong positive correlation between measures of the SARS-CoV-2-specific cellular immune response (QF whole genome and Flow S1 CD 8+; Rho 0.80, p<0.001). On the other hand, antibody levels in our patient cohort showed very weak negative or no correlation at all with the cellular immune response against SARS-CoV-2 (**Figure 2**).

### Associations between risk factors, SARS-CoV-2 specific immune response, and mortality

Only obesity was associated with marginal excess mortality in the patient group from well-established risk factors including obesity, hypertension, diabetes, and smoking using long-rank tests Kaplan Meier curves. Their respective long-rank tests are shown in **Supplementary Figure 2**.

**Figure 3:**
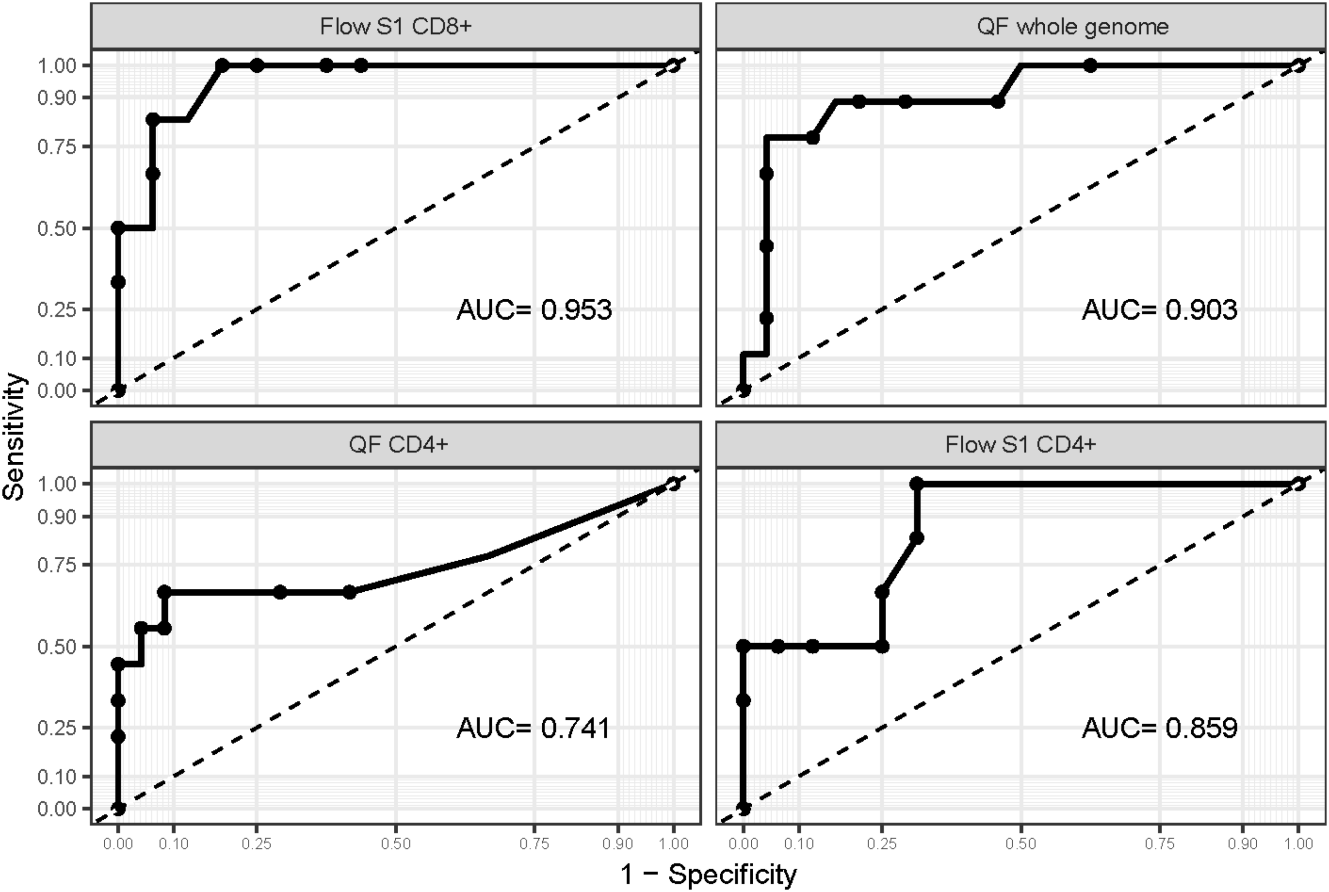
ROC curves are drawn for markers of the cellular immune response. ROC curves were drawn to ascertain the ideal cutoff point for each marker of the cellular immune response. AUC values suggest that Flow S1 CD8+ and QF whole genome levels measured at ECMO initiation can help identify patients at risk with high accuracy.

**Figure 4:**
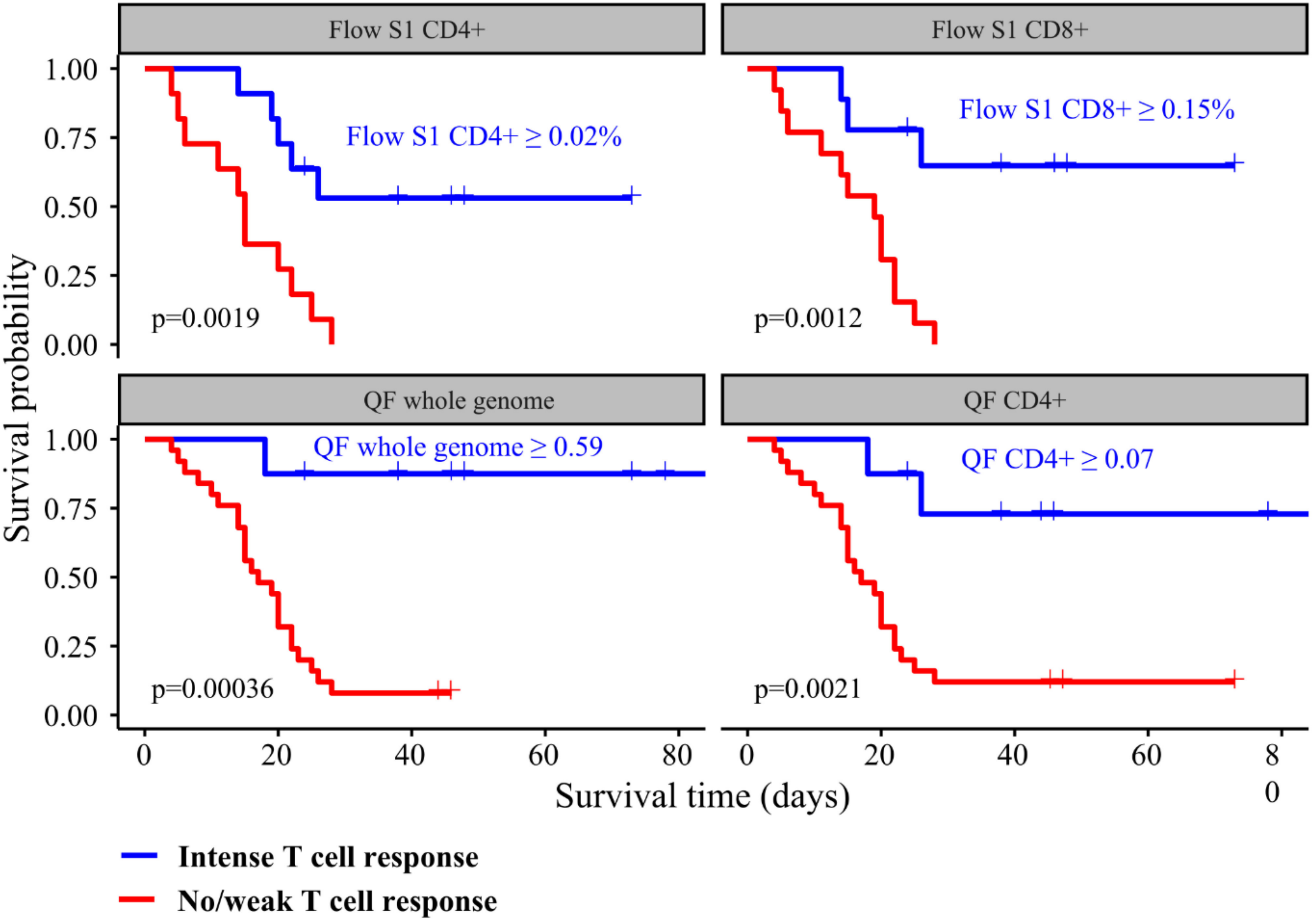
Kaplan Meier curves showing the associations between T cell response intensity and all-cause mortality among critically ill COVID-19 patients. Patients were categorized according to the intensity of their immune response.

We performed ROC analysis to ascertain the ideal cutoff values for immune parameters to inform patient outcomes. Immune samples taken during VV-ECMO cannulation showed that a higher probability of survival was observed in patients with markers indicating higher T cell response (Flow S1 CD8+ ≥ 0.15%, Flow S1 CD4+ ≥ 0.02%, QF CD4 ≥ 0.07, QF whole genome ≥ 0.59), as shown in **Figure 3**. Indeed, all the patients who had no detectable CD4+ and CD8+ T lymphocyte response by functional flow cytometry and QantiFeron assay at the initiation of ECMO treatment passed away. Kaplan Meier curves with their respective long rank tests depicting these associations are shown in **Figure 4**.

Finally, we performed Cox proportional hazard regression models to study the magnitude of the association between clinical and immune markers and mortality. In univariate analysis, only BMI, RV failure, QF whole genome T cell level, and Flow S1 CD8+ T cell level were associated with mortality (**Table 4**). Based on the results from the univariate analysis and biological plausibility, we assembled two multivariate models to determine the relationship between these variables as shown in **Tables 5A**, **B**. Results from our multivariate models showed that a decreased T cell activity measured by flow cytometry associated with mortality independently of BMI and RV failure. **Supplementary Table 3** shows the association between clinical factors arising after ECMO cannulation.

**Table 4:** Examining the association between clinical factors and in-hospital mortality *via* univariate Cox proportional hazard regression in severely ill SARS-CoV-2 patients requiring ECMO support.

	beta	HR (95% CI for HR)	wald.test	p.value
White blood cell count	0.019	1 (0.98-1.1)	0.9	0.34
Lymphocyte count	0.226	1.3 (0.64-2.4)	0.43	0.51
Lymphocyte percentage	-0.028	1 (0.90-1.05)	0.53	0.47
Age	0.017	1.02 (0.99-1.05)	1.63	0.20
BMI	0.088	1.09 (1.01-1.18)	5.31	0.02*
NCP IgG	0.078	1.08 (0.92-1.27)	0.89	0.35
NCP IgM	0.008	1.01 (0.91-1.12)	0.02	0.88
SP1 IgA	0.010	1.01 (0.91-1.13)	0.03	0.86
Roche spike-RBD Ig (G+A+M)	0.0002	1.00 (1.00-1.00)	1.47	0.19
QF CD4+	-7.47	0.001 (0.00-2.31)	3.10	0.08
QF CD4+ and 8+	-9.91	0.00 (1.4*e-10*-17.51)	2.31	0.13
QF whole genome	-1.39	0.25 (0.07-0.90)	4.52	0.03*
Flow S1 CD4+	-4.73	0.01 (0.00-1.87)	3.00	0.08
Flow S1 CD8+	-7.99	0.0003 (0.00-0.25)	5.65	0.02*
Flow NC CD4+	-40.96	1.62*e-18* (1.12*e-38* -233.94)	2.99	0.08
Flow NC CD8+	-1.38	0.25 (0.01 -8.11)	0.61	0.43
Obesity	0.76	2.13 (0.97-4.66)	3.57	0.06
Hypertension	0.38	1.47 (0.63-3.40)	0.80	0.37
Smoking	0.26	1.3 (0.39-4.4)	0.18	0.67
Diabetes	-0.33	0.72 (0.17-3.03)	0.20	0.65
Renal failure prior to ECMO cannulation	-0.04	0.96 (0.40-2.29)	0.01	0.93
Right ventricular failure prior to ECMO cannulation	0.94	2.55 (1.14-5.75)	5.15	0.02*
Bacterial superinfection	-0.09	0.92 (0.42-1.99)	0.05	0.83
Time from COVID-19 symptoms to ECMO	-0.0001	1 (0.91 – 1.10)	0.00	0.99
Time from IMV to ECMO	0.03	1.03 (0.90 – 1.19)	0.20	0.66
Pentaglobin	-0.62	0.53 (0.23 – 1.24)	2.13	0.14

Results show the unadjusted beta coefficients, the effect sizes (given as hazard ratios), and statistical significance for each of the variable in relation to survival. Each factor is assessed through a separate univariate Cox proportional hazard regression model, each row depicting the results from a separate model. Asterix (*) shows significant results.

**Table 5A:** Multivariate Cox proportional hazard regression model 1.

	beta	HR (95% CI for HR)	wald.test	p.value
BMI	0.002	1.00 (0.89-1.12)	0.001	0.97
Right ventricular failure prior to ECMO cannulation	-0.06	0.9 (0.31-2.84)	0.01	0.92
Flow S1 CD 8+	-8.07	0.0003 (0.00-0.85)	4.00	0.04*

Asterix (*) shows significant results.

**Table 5B:** Multivariate Cox proportional hazard regression model 2.

	beta	HR (95% CI for HR)	wald.test	p.value
BMI	0.04	1.04 (0.97 -1.13)	1.24	0.27
Right ventricular failure prior to ECMO cannulation	0.43	1.50 (0.66 -3.61)	1.01	0.32
QF whole genome	-1.07	0.34 (0.09 -1.27)	2.55	0.11

Results from the two multivariate Cox proportional hazard regression models. Each table shows a separate model. Both models were assembled based on the results from the univariate models and biological plausibility.

## Discussion

### Summary of findings

We assessed the cellular and humoral immune response to SARS-CoV-2 infection and its associations with mortality in a cohort of 35 unvaccinated, critically ill patients requiring ECMO support. Here, we demonstrated for the first time that SARS-CoV-2 specific cellular immune response measured at the time of VV-ECMO cannulation identifies patients who have a higher probability of survival, independent of BMI and RV failure. Our patients generally presented with a high burden of comorbidities, and 26% (9/35) of them were discharged alive. It is noteworthy that the burden of risk factors, comorbidities, and the markers of humoral immune response did not differ significantly between surviving and deceased patients.

### Comparison with the literature

Our single-center study provides new data on severely ill COVID-19 patients, assessing the effectiveness of ECMO support in SARS-CoV-2 patients. Our patients were somewhat younger and more likely to be women compared to the patient population of the International Extracorporeal Life Support Organization (ELSO) Registry (19). Of note, some recent meta-analyses suggested a much lower in-hospital mortality rate for ECMO patients after COVID-19 (39%) (20). Factors in our study that might have driven this relatively high mortality rate were the delayed ECMO referral in our patient cohort as well as the high burden of renal failure, right-sided heart failure, and bacterial superinfection found in our subset at the initial assessment after ECMO support initiation. Importantly, all the patients were unvaccinated in our study. At the same time, 26% of the patients were saved using the device therapy, underscoring the importance of promoting education for adequate patient referral and timely action among referring clinicians.

The key finding of our study is the distinctive immune response between survivors and deceased critically ill COVID-19 patients requiring ECMO support. Previous studies have shown dysregulation of innate and adaptive immune cell compartments in patients with convalescent COVID-19, suggesting an association between CD4+ and CD8+ T cell response and disease severity (21–24). However, to the best of our knowledge, we are the first to report the potential protective role of the strong T cell response against in-hospital mortality among severely ill COVID-19 patients requiring ECMO support. The role of the T cell response in controlling infection or contributing to pathology has been extensively studied since the beginning of the pandemic (9, 25, 26). Th1 response is required to clear the infection and both the lack- and over-activation of Th1 response is associated with poor prognosis. Although underlying immunological mechanisms are not fully understood yet, there is increasingly more evidence in support of the importance of a Th1-geared cellular immune response against SARS-CoV-2, which is further supported by our results.

All patients tested positive by serological assays including selective anti-SARS-CoV-2 NCP IgG. Our results showed that a strong antibody response by virus-neutralizing antibody activity underlies the severe clinical symptoms detected 3 weeks after the onset of several COVID symptoms. Moreover, our deceased patients had similarly high levels of specific anti-SARS-CoV-2 NCP IgG measured by ELISA as survivors. Here, we detected a highly accelerated B cell immune response by antibody level overshoot (evidenced by the very high antibody levels themselves). Alongside this, deceased patients presented no or weak T cell response in peripheral blood, which altogether suggests that a shift in the balance between cellular and humoral immune response toward the latter in critically ill patients is associated with a poor outcome. Of note, none of the patients had a history of T cell immunodeficiency that would account for the results. These results are in line with findings from Wen et al. among moderately and severely ill COVID-19 patients (27). Furthermore, in previous reports, the absence of SARS-CoV-2-specific CD4^+^ T cells was associated with severe or fatal COVID-19 (28, 29).

Cytokine profiling in our patient population could not be expected to give unbiased results due to the adjuvant cytokine depletion *via* CytoSorb applied routinely as part of local ECMO protocol. Antibody production and T cell activation, measured in our study, can still be considered as downstream readouts of the same Th1/Th2 axis. From this perspective, our observations provide further, clinical evidence on the importance of cellular immune responses in COVID mortality risk.

Based on our study, the patients who had cellular immune response levels above the cutoff points had a higher probability to survive a severe COVID-19 infection on ECMO treatment. Exactly how these cell types contribute to the promotion or prevention of pathology remains to be fully elucidated (26, 30). However, our results offer novel avenues of research to improve the evaluation of seriously ill COVID-19 patients undergoing VV-ECMO support, with additional opportunities to predict the outcomes, and potentially change our clinical practice in the future. It is also essential to note that a comprehensive cost-effectiveness analysis is a crucial next step to solidify these findings and affirm the practical applicability and usefulness of such therapeutic interventions in clinical settings.

### Limitations

Our results are limited by the following factors: first, this is a single-center study with a small sample size of a unique cohort, which may have led to the underestimation of our hazard ratios (regarding age or other important comorbidities). Second, ECMO is a highly resource-intensive therapeutic procedure and its accessibility is limited by high cost, staffing, and training requirements. Third, patients were referred to our institution by other care providers, therefore we cannot exclude referral bias driven by referring physicians’ experience or ECMO-specific training level. Lastly, at the time of our study, there was no uniform referral system for ECMO regarding at what point in the severity of the disease a patient was referred for ECMO (considering the current metabolic status, ventilation parameters, and fluid therapy).

### Clinical applications

Selecting patients for ECMO support is notoriously difficult in times of COVID surges and limited supplies. Aiding the physicians to make certain decisions grants valuable insight into the selection procedure of an appropriate and effective therapy. The key potential implementation of this study is hypothesis generation for further research. Assessment of T cell response among patients referred to ECMO might be beneficial in terms of decision-making and triage of critically ill COVID-19 patients in the future. Based on our findings, further investigations could help determine if the VV-ECMO guideline should include the T cell status evaluation as a predictive factor in the criteria for the VV-ECMO initiation for seriously ill COVID-19 patients.

## Conclusions

Our data could help in the selection of seriously ill COVID-19 patients undergoing VV-ECMO support, with additional opportunities to predict the outcomes, changing our clinical practice in the future. Based on our findings, further investigations could help determine if the VV-ECMO guideline should include the T cell status evaluation as a predictive factor in the criteria for the VV-ECMO initiation for seriously ill COVID-19 patients.

## Data availability statement

The original contributions presented in the study are included in the article/**Supplementary Material**. Further inquiries can be directed to the corresponding author.

## Ethics statement

The studies involving human participants were reviewed and approved by National Public Health Center. The patients/participants provided their written informed consent to participate in this study.

## Author contributions

ZU, HV, and GN contributed to the conceptualization of the study. ZS, EvK, LF, AV, NK, EnK, DN, EN, and ZU supported the investigation and data collection. ZU contributed to the data curation. LS supported the data curation. TS and LS performed the statistical analysis. ZU and LS wrote the original manuscript. HV and BM contributed to the funding acquisition. All co-authors read and revised the manuscript. All authors contributed to the article and approved the submitted version.

## Glossary

**Table:**

ARDS	acute respiratory distress syndrome
BMI	Body Mass Index
COPD	Chronic Obstructive Pulmonary Disease
COVID-19	coronavirus disease 2019
CRRT	Continuous Renal-Replacement Therapy
ECMO	extracorporeal membrane oxygenation
ELSO	Extracorporeal Life Support Organization
FIO2	fraction of inspired oxygen
ICU	intensive care unit
IFN-γ	interferon-γ
IL-6	interleukin-6
IMV	invasive mechanical ventilation
IQR	InterQuartile Range
NAbs	neutralizing antibodies
NC	nucleocapsid
PaO2	partial pressure of arterial oxygen
QF	QuantiFeron
RBD	receptor binding domain
RV	right ventricle
SARS-CoV-2	severe acute respiratory syndrome coronavirus 2
S1	spike protein 1
S-specific	spike specific
V-V	venovenous
V-V ECMO	venovenous extracorporeal membrane oxygenation
WBC	white blood cell
WHO	World Health Organization
