# Supplementary material for: T cell immune response predicts survival in severely ill COVID-19 patients requiring venovenous extracorporeal membrane oxygenation support
Source: Front Immunol. 2023 Aug 1;14:1179620. doi: 10.3389/fimmu.2023.1179620 (PMC10433181; doi:10.3389/fimmu.2023.1179620)
Supplement: Supplementary file 1 [file DataSheet_1.docx]

**Supplementary Table 1:** Measures of the SARS-CoV-2 specific immune response

| Test name | Test target | Measured variables and cut off | Measuring device | Measuring principle/ method | Reference |
| --- | --- | --- | --- | --- | --- |
| Humoral immun response | | | | | |
| RocheElecsys ELISA test (Roche Diagnostics International Ltd, Switzerland) | Antibodies specific to SARS-CoV-2 spike (S) protein receptor binding domain (RBD) in human serum and plasma | IgG, IgA, IgM against SARS CoV 2 (quantitative)  Limit of quantification: 0.4 U/ml.  <0.8 U/ml – negative  >0.8 U/ml – positive | Elecsys Anti-SARS-CoV-2 S immunoassay (Roche Diagnostics International Ltd, Switzerland) on a Cobas e6000 instrument | Double-antigen sandwich principle using electrochemiluminescence for quantitative determiation of antibodies. | (Riester et al. 2021) |
| Euroimmun ELISA test | Humoral immunresponse –  antibodies produced against different antigens of the virus: nucleocapsid/spike proteins | Anti-SARS-CoV-2 QuantiVac ELISA (IgG):  The results are given in relative units (RU/ml).  <8 RU/ml – negative  8 – 11 RU/ml – borderline  >11 RU/ml – positive  Upper limit of quantification: 120 RU  Anti-SARS-CoV-2 ELISA (IgA):  The results are given in ratio (extinction of the sample/extinction of calibrator).  <0.8 – negative  0.8 – 1.1 – borderline  >1.1 – positive  Anti-SARS-CoV-2 NCP ELISA (IgG and IgM):  The results are given in ratio (extinction of the sample/extinction of calibrator).  <0.8 – negative  0.8 – 1.1 – borderline  >1.1 – positive | Sample dilution was performed manually, further steps were carried out automatically using an Elite Lite (DAS, Italy) device. | Enzyme immunoassay provides semiquantitative in vitro determination of human antibodies of different immunoglobulin classes. | (Beavis et al. 2020; Dolscheid-Pommerich et al. 2022; Pieri et al. 2020) |
| Cellular immun response | | | | | |
| QuantiFERON SARS-CoV-2 ELISA – IGRA (Interferon-Gamma Release Assays) test | Antigen tubes targetted to different cellular markers. Blood collection tubes coated with specific SARS-CoV-2 peptides pool from spike antigen (S1 S2 RDB). Blood collection tubes coated with specific SARS-CoV-2 peptides pool from spike and additional peptides issued (N (nucleocapsid) and M (M protein) domains) from the full genome of the SARS-CoV-2 virus. | QF Ag1 stimulates CD4+ T cells  QF-Ag2 stimulates both CD4+ CD8+ T cells with S1/S2 RBD antigen  QF-Ag3 stimulates both CD4+ CD8+ T cells with spike and additional peptides, including nucleoprotein  Results:  >0.15 – 0.2 IU/ml – positive | QuantiFERON ELISA (QIAGEN Group) | Based on the production of interferon gamma (IFN-γ) by lymphocytes in peripheral blood. The QF Ag1 stimulates CD4+ T cells, QF-Ag2 stimulates both CD4+ CD8+ T cells with S1/S2 RBD antigen, while QF-Ag3 stimulates both CD4+ CD8+ T cells with spike and additional peptides, including nucleoprotein. After 16-24 hours of incubation at 37°C, the IFN-γ produced during the cell activation was measured from the separated plasma samples by QuantiFERON ELISA | (Krüttgen et al. 2020) |
| Functional flow cytometry | Virus specific T cells were detected by functional flow cytometry based on IFN-γ secretion. | Cut-off value was set as 0.02% IFNγ+ T cells (minimum 50 events). | Peptivator SARS-CoV-2 Protein S1, S and N  (Miltényi Biotec)  #130-127-585  #130-127-586  #130-127-583  Cytostim  #130-092-172  10ul IFNγ-PE and 10ul CD3-VioBlue /CD4APC/ CD8FITC/ CD14-Per-CP/ CD20-PerCP  Miltenyi Biotec, Rapid Cytokine Inspector CD4/CD8 Kit  #130-097-343  Rapid Cytokine Inspector Anti-IFNγ-PE # 130-097-600  Cytoflex flow cytometer (Beckman Coulter) | Human peripheral blood mononuclear cells isolated by density gradient centrifugation and mononuclear cells were stimulated with 6nmol Peptivator SARS-CoV-2 Protein S1, S for two hours at 37⁰C and 5% CO2, then incubation continued in the presence of Brefeldin-A for 4 hours at 37⁰C and 5% CO2. Unstimulated samples without Peptivators were used as negative controls, and samples stimulated with Cytostim served as positive controls. For surface and intracellular staining cells were incubated with a staining mix of antibody cocktail for 10 minutes at room temperature, then Inside Fix and Inside Perm reagents were used for cell fixation and permeabilization. After cell wash and centrifugation, 500 000 cells were acquired from each sample using flow cytometer. Analysis was performed according to Miltenyi Biotec gating strategy: Doublets (FSC-H/FSC-A), CD19+ B cells and CD14+ monocytes were excluded. T cells were identified by CD3 positivity and scatter characteristics (FSC/SSC). Ratio of INF-γ positive virus specific T cells was determined within the CD4+ and CD8+ T cell population separately, compared to negative control. | ??? |

**Supplementary Figure 1:**


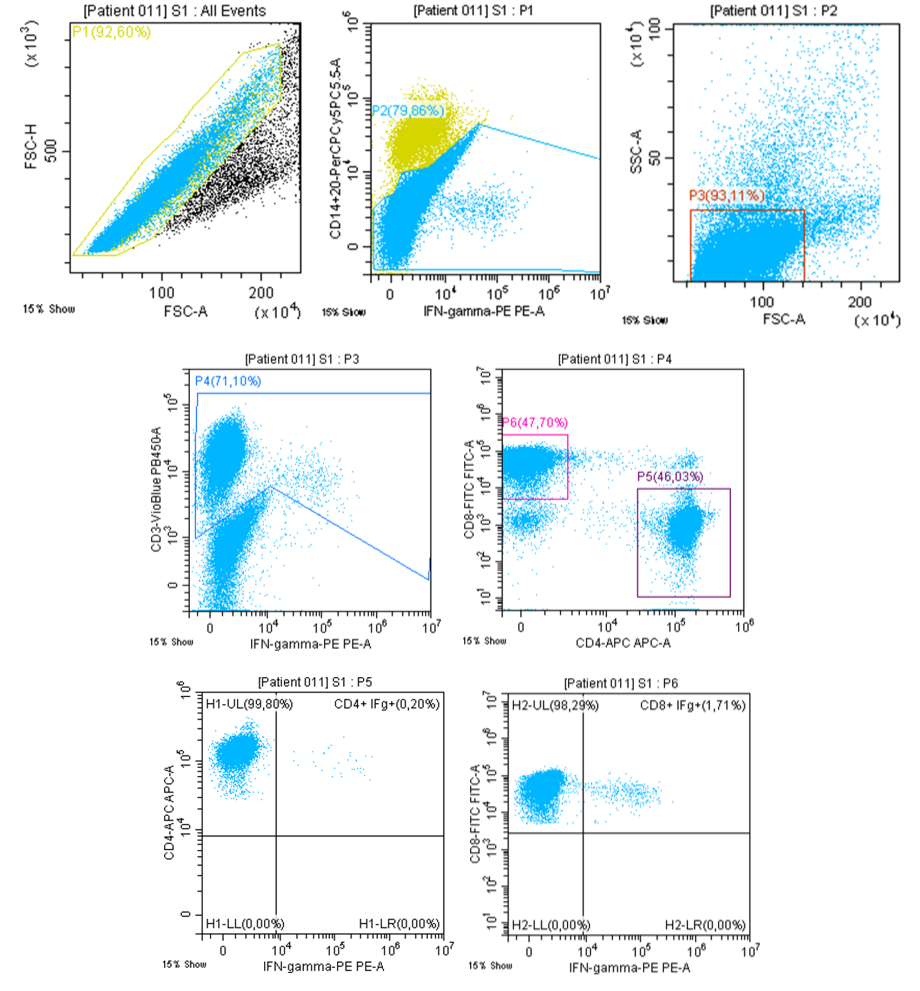


**Footnote:** Patient sample with detectable SARS-CoV-2 specific T cells (VST positive sample). Sequential gating strategy by Milteny. P1: Single cells from PBMCs.  P2: CD20 negative and CD14 negative cells. (Exclusion of B cells and monocytes). P3: Lymphocytes with low FSC/SSC characteristics.  P4: CD3+ T cells. P5: CD4+ T cells, P6: CD8+ T cell. Percentage of IFNg positive T cells: 0.20% and 1,71% virus specific T cells were identified within the CD4+ and CD8+ T cell population, respectively.

**Supplementary Table 2:** Data completeness documentation

| Variable | Number of records | Considered in survival analysis |
| --- | --- | --- |
| Age | 35(100%) | Yes |
| Sex | 35(100%) | Yes |
| BMI | 35(100%) | Yes |
| Obesity status | 35(100%) | Yes |
| Hypertension status | 35(100%) | Yes |
| Diabetes status | 35(100%) | Yes |
| Smoking status | 35(100%) | Yes |
| Peripartum infection status | 35(100%) | Yes |
| Hypothyreosis status | 35(100%) | No |
| Hasimoto status | 35(100%) | No |
| Arthritis status | 35(100%) | No |
| Prevalent myocardial infarction staus | 35(100%) | No |
| Prevalent atrial fibrillation status | 35(100%) | No |
| Prevalent chronic obstuctive pulmonary diasease stauts | 35(100%) | No |
| White blood cell count | 35(100%) | Yes |
| Lymphocyte count | 35(100%) | Yes |
| Lymphocyte present | 35(100%) | Yes |
| NCP IgG | 33(94%) | Yes |
| NCP IgM | 33(94%) | Yes |
| SP1 IgA | 33(94%) | Yes |
| QF CD4 | 33(94%) | Yes |
| QF CD4 8 | 33(94%) | Yes |
| QF AG3 | 33(94%) | Yes |
| RocheIgG | 31(89%) | Yes |
| Flow CD4 | 22(63%) | Yes |
| Flow CD 8 | 22(63%) | Yes |
| Flow NC CD4 | 22(63%) | Yes |
| Flow NC CD8 | 22(63%) | Yes |
| Flow S CD4 | 14(40%) | No |
| Flow SCD8 | 14(40%) | No |
| Sp1 IgG | 11(31%) | No |


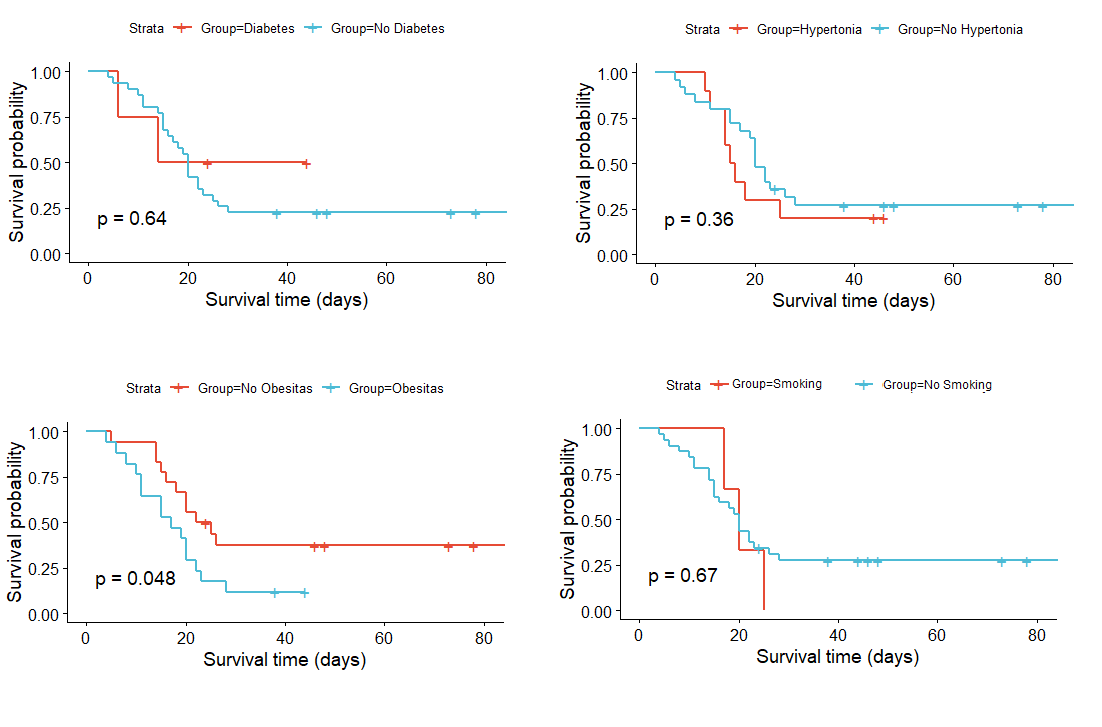
**Supplementary Figure 2:** Kaplan Meier curves with their respective logrank tests illustrating the difference between the mortality of study participants with or without a given risk factor

**Supplementary Table 3:** The association between clinical factors arising after ECMO cannulation and mortality

|  | beta | HR (95% CI for HR) | wald.test | p.value |
| --- | --- | --- | --- | --- |
| Renal failure after ECMO cannulation | 0.55 | 1.73 (0.79-3.77) | 1.89 | 0.17 |
| Liver failure after ECMO cannulation | 0.68 | 2 (0.74-5.26) | 1.82 | 0.18 |
| Multi organ failure after ECMO cannulation | 15.32 | 4510935.16 (5.7423E-249 - 3.5436E+261) | 0.002 | 0.96 |

**Footnote:** Univariate analysis showing the assocation between organ failure detected after ECMO cannulation and in-hospital morality.
